# Molecular characterization of an outbreak of enterovirus-associated meningitis in Mossel Bay, South Africa, December 2015–January 2016

**DOI:** 10.1186/s12879-018-3641-4

**Published:** 2018-12-29

**Authors:** Heidi Smuts, Sarah Cronje, Juno Thomas, Delene Brink, Stephen Korsman, Diana Hardie

**Affiliations:** 1 Division of Medical Virology, Department of Pathology, Faculty of Health Sciences, University of Cape Town, South Africa and the National Health Laboratory Service, Anzio Road, Observatory, Cape Town, 7925 South Africa; 2 Life Bay View Private Hospital, Ryk Tulbach Street & Alhof Drive, De Nova, Mossel Bay, 6506 South Africa; 3 Centre for Enteric Diseases, National Institute for Communicable Diseases, 1 Modderfontein Road, Sandringham, Johannesburg, 2130 South Africa; 4 PathCare George Laboratory, 1 Gloucester Avenue, George, 6529 South Africa

**Keywords:** Aseptic meningitis, Coxsackie virus A9, Outbreak, South Africa

## Abstract

**Background:**

Human enteroviruses (HEVs) are common causal agents of aseptic meningitis in young children. Laboratory and syndromic surveillance during December 2015 and January 2016 noted an unusually high number of paediatric aseptic meningitis cases at a hospital in Mossel Bay, Western Cape Province, South Africa. HEV was detected in clinical samples, prompting an outbreak investigation.

**Methods:**

Epidemiological investigations were conducted to ascertain possible linkage between cases. Amplification, sequencing and phylogenetic analysis of the 5’UTR and VP1 regions was undertaken to determine the HEV serotype associated with the outbreak as well as other cases of aseptic meningitis in the area in the preceding 6 weeks.

**Results:**

Over the 2-month period, 63 CSF samples were available for testing. A total of 43 outbreak cases (68.3%) were observed, and the 26 (60.5%) that could be typed were coxsackie virus A9 (CVA9). Children attending three crèche facilities were epidemiologically linked, accounting for 60.5% (26/43) of the CVA9 cases. The majority of patients were under 10 years of age (55/63, 87.3%) and there was a male predominance (66%). Nucleotide sequence analysis of the 5’UTR and VP1 regions identified 2 lineages of CVA9 co-circulating during the outbreak, although the VP1 capsid protein sequence was identical as all nucleotide differences were synonymous. There was a unique isoleucine at position 64 and all outbreak viruses had a valine to threonine change in the hypervariable BC loop of VP1. Other HEV types circulating in the preceding period were echovirus 30 (*n* = 4), echovirus 5 (*n* = 3) and 1 each of echovirus 6, echovirus 9 and echovirus 15.

**Conclusion:**

CVA9 was identified as the pathogen responsible for the large outbreak of aseptic meningitis, with 2 distinct co-circulating lineages.

**Electronic supplementary material:**

The online version of this article (10.1186/s12879-018-3641-4) contains supplementary material, which is available to authorized users.

## Background

Human enteroviruses (HEVs) are ubiquitous and cause an array of clinical syndromes ranging from asymptomatic infections, in about 80% of cases, to more severe illnesses including respiratory infection, acute haemorrhagic conjunctivitis, hand- foot-and-mouth disease, neonatal sepsis, myocarditis, paralytic disease, encephalitis and aseptic meningitis [1, 2]. The virus is spread from person to person most commonly via the faecal-oral route and less frequently through respiratory droplets [3, 4]. Transmission often occurs within the family, in schools, hostels and chronic care facilities [1, 4]. Community and nosocomial spread is also reported, particularly where there is overcrowding [3, 5].

Most HEV infections occur in the summer/autumn months in temperate climates and throughout the year in tropical regions [4, 6]. Usually only a few serotypes predominate in each HEV season and these can lead to outbreaks of a localised or more widespread nature.

HEVs belong to the family *Picornaviridae*, genus *Enterovirus* and are classified into 4 species (A, B, C and D) based on neutralizing assays, sequence data and analysis of the virus capsid VP1 gene [7–9]. The VP1 capsid protein is exposed on the outer surface of the virion, has specific epitopes, particularly in the BC loop, that elicit type-specific neutralising antibodies and are primarily responsible for defining the serotype [1, 6, 10–12]. Laboratory diagnosis using molecular technologies enables rapid detection of HEV and has largely replaced the more labour intensive virus culture and isolation methods. Reverse transcription PCR (RT-PCR) diagnostic methodologies target the most conserved region of the HEV genome, 5′ untranslated region (5’UTR), while typing for the investigation of outbreak and clinical disease associations usually targets the VP1 gene.

Aseptic meningitis is a self-limiting illness occurring in adults and children, with neonates most at risk of severe systemic illness. HEVs are the most common causal agent and association is inferred by the detection or isolation of virus from the cerebrospinal fluid [13].

Numerous viral meningitis outbreaks associated with HEVs have been reported in South Africa. The most comprehensive study from Cape Town during 1981–1989 identified 5 major episodes involving over 1400 cases with echovirus 4, echovirus 9 and coxsackie virus A9 (CVA9) most commonly identified [14]. Other reports include an outbreak of echovirus 3 at a summer camp in 2001 involving 15 children [15], 9 cases of enterovirus 84 in the small town of Ladismith in 2009 [16] and an outbreak of echovirus 4 in 23 children from the Tshwane region in 2010–2011 [17].

This study reports the epidemiology of a large outbreak of aseptic meningitis associated with enteroviruses in Mossel Bay, Western Cape Province, South Africa from December 2015–January 2016.

## Objectives

The objective of this study was to determine the molecular epidemiology of HEVs in the CSF of patients presenting with aseptic meningitis in Mossel Bay, South Africa, over a 2-month period.

## Methods

### Patients and samples

Mossel Bay is coastal town in the Western Cape Province, South Africa, located midway between Cape Town and Port Elizabeth. There is one municipal and one private hospital and numerous educational and crèche facilities. During the study period schools closed on 12th December 2015 for annual summer vacation and reopened on 13th January 2016.

In mid-January 2016 local healthcare workers noticed an increase in young children presenting at a private sector hospital (Life Bay View Private Hospital) with clinical manifestations and laboratory findings suggestive of aseptic meningitis. The increase was from 3 to 7 cases weekly preceding the outbreak to 4–6 cases daily during the outbreak period. Cerebrospinal fluid (CSF) samples were collected from hospitalised patients and tested at a private laboratory (PathCare, in George), where an HEV was identified as the causative pathogen. The Western Cape Department of Health was notified of the suspected outbreak, prompting an epidemiological investigation to ascertain possible links between cases, implementation of control measures, and health promotion activities. Demographic data was collected on all HEV-positive patients hospitalised with symptoms of aseptic meningitis from 1st December 2015 to the first week in February 2016 in order to place the outbreak in context.

CSF samples from aseptic meningitis cases were referred to the PathCare George laboratory and screened using the Meningitis/Encephalitis (ME) Panel (BIOFIRE® FILMARRAY®) which detects 14 relevant bacterial, viral and yeast pathogens. Samples that were positive for HEV and still stored at the PathCare George laboratory were sent to the NHLS Molecular Virology diagnostic laboratory in Cape Town for HEV typing.

### Case definitions

A confirmed case of aseptic meningitis was defined as any person admitted to the Life Bay View Private Hospital in Mossel Bay between 1st December 2015 and 3rd February 2016 with a history of acute onset of severe headache, nausea and/or vomiting and fever with compatible laboratory findings on CSF analysis.

### RNA extraction

Total nucleic acid was extracted from 500 μl CSF using the NucliSENS easyMag (bioMerieux SA, Lyon, France) and eluted in 50 μl elution buffer.

### HEV typing by VP1, VP2 and 5’UTR amplification

cDNA was generated with random primers using the RevertAid First Strand cDNA synthesis kit (Thermo Scientific, Lithuania), as per manufacturer’s instructions.

Species-specific nested PCRs were used to amplify a region of the VP1 gene of EV-A and EV-B using primers from McWillliam Leitch et al. [18], while the VP2 gene was used to detect EV-C [19], generating PCR fragments of 784 bp, 1085 bp and 406 bp, respectively. Samples that were still negative after species- specific amplification were investigated by amplification of a 498 bp fragment of the 5’UTR region [18]. The PCR was performed in a 50 μl reaction containing 5 μl cDNA, 15 mM Tris-HCL (pH 8), 50 mM KCl, 1.5 mM MgCl_2_, 0.2 mM dNTPs (ABgene, Epsom, UK), 50 pmol of primers and 1.5 U Supertherm Taq polymerase (JMR Holdings, Kent, UK). Amplification was as follows: 95 °C for 3 min, 40 cycles of 95 °C for 15 s, 50 °C for 25 s and 72 °C for 35 s followed by 72 °C elongation for 7 min. Nested amplification was the same, but with an annealing temperature of 55 °C. PCR products were visualised by electrophoresis through a 2% agarose gel, ethidium bromide staining and UV illumination.

### Sequencing

PCR products were cleaned up using the Zymoclean Gel DNA Recovery kit (Zymo Research Corp. Irvine, California, USA) and sequenced directly with the BigDye terminator cycle sequencing kit (Applied Biosystems, Foster City CA, USA) using PCR-specific primers. Sequences were confirmed to be of HEV origin using BLASTn [20] and the Enterovirus Genotyping Tool Version 0.1 [21]. Sequences obtained were aligned with reference sequences obtained from the GenBank database and the NIAID Virus Pathogen Database and Analysis Resource (ViPR) [22] through the website “http://www.viprbrc.org/” using BioEdit version 7.2.5 [23]. Additional HEV sequences from Cape Town (NHLS Virology Molecular Diagnostic Laboratory) and Johannesburg (National Institute for Communicable Diseases, NICD) were also included. Neighbour-joining phylogenetic trees were constructed in MEGA 6.06 using the maximum-likelihood algorithm with 1000 bootstrap re-sampling [24]. Highlighter plots were generated by Highlighter [25] using 5’ UTR and VP1 aligned nucleotide and amino acid sequences to visualize individual sequence polymorphisms.

### Nucleotide accession numbers

The 5’UTR and VP1 sequences reported in this study were deposited in the GenBank sequence database under accession numbers MH558061 - MH558083 and MH558084 - MH558112, respectively. The raw nucleotide data is also available (Additional files 1 and 2).

## Results

Epidemiological investigation during the 2-month period (1st December 2015 to 3rd February 2016) identified 63 laboratory-confirmed cases of HEV meningitis (Table 1). Forty-three (68.3%) cases were associated with the outbreak as these could either be epidemiologically linked, and/or occurred after the reopening of schools on 13th January 2016. In addition, where typing was successful, CVA9 was linked to these outbreak cases (26/43, 60.5%) (see below). Accordingly, it was presumed that the 43 cases of the outbreak were caused by CVA9. In the remaining 20 cases no epidemiologically linkage could be established. These cases probably represented sporadic cases of aseptic meningitis, with different HEV types, occurring during the summer season when aseptic meningitis caused by HEVs is common. All patients were admitted to hospital. The demographic features of the laboratory-confirmed cases are shown in Table 1.

**Table 1:** Demographics of the HEV-positive aseptic meningitis cases

Patient	Age Range (years)	Place of permanent residence	Epidemiological link	HEV serotype
1501	0–4	NK	No school involvement	
9525	0–4	Mossel Bay	No school involvement	
9365	0–4	Mossel Bay	Unknown	E9
0784	0–4	Pretoria	No school involvement	E30*
0780	0–4	Mossel Bay	No school involvement	
0785	0–4	Mossel Bay	Unknown	E6*
1502	0–4	NK	No school involvement	
0838	10–14	Witbank	No school involvement	E5
0847	5–9	Pretoria	No school involvement	E5
0864	0–4	Mossel Bay	No school involvement	
1503	10–14	NK	No school involvement	
1104	0–4	Middelburg	No school involvement	E5
0947	5–9	Pretoria	Unknown	
2083	5–9	Riversdal	Unknown	CVA9
2149	10–14	Kimberley	Unknown	E30*
2314	0-4	Mossel Bay	Unknown	CVA9/CVB3*
2319	0-4	Mossel Bay	Unknown	
2446	5–9	Douglas	Unknown	E15*
2572	5-9	Jan Kempdorp	Unknown	
2407	5–9	Bloemfontein	Unknown	E30
3965	10–14	Mossel Bay	Unknown	CVA9
4103	0–4	Mossel Bay	Unknown	
4751	0–4	Mossel Bay	Unknown	CVA9
5012	0–4	Mossel Bay	A crèche	CVA9
5018	0–4	Mossel Bay	Unknown	CVA9
4809	0–4	Mossel Bay	A crèche	CVA9
1601	0–4	Mossel Bay	A crèche	
4969	0–4	Mossel Bay	Unknown	CVA9/E16*
5081	5-9	Mossel Bay	Unknown	CVA9
5084	0–4	Mossel Bay	Unknown	CVA9
1602	10–14	Mossel Bay	C crèche	
5114	0–4	Mossel Bay	B crèche	CVA9
5146	0–4	Mossel Bay	B crèche Sibling of 4938	CVA9
5064	0–4	Mossel Bay	B crèche	CVA9/E16*
1603	0–4	NK	A crèche	
5088	0–4	Mossel Bay	B crèche	CVA9
5100	0–4	Mossel Bay	B crèche	
5112	0–4	Mossel Bay	B crèche	CVA9/CVB3*
5145	0–4	Mossel Bay	B crèche	CVA9
5157	0–4	Mossel Bay	B crèche	
1604	0–4	Mossel bay	B crèche	
5110	0–4	Mossel Bay	B crèche Child of 5470	CVA9
4929	0–4	Mossel Bay	B crèche	CVA9
4938	0–4	Mossel Bay	Sibling of 5146	CVA9
4962	0–4	Mossel Bay	B crèche	CVA9/CVB3*
4965	0–4	Mossel Bay	C crèche	CVA9
1605	5–9	Mossel Bay	C crèche	
5408	10–14	Mossel Bay	Unknown	CVA9/ E16*
5411	10–14	Mossel Bay	Unknown	
5433	0–4	Mossel Bay	Unknown	CVA9/CVB3*
5470	30-40	Mossel Bay	Parent of 5110	CVA9/CVB3*
5575	0–4	Mossel Bay	Unknown	CVA9/ CVB3*
5486	5–9	Mossel Bay	Unknown	
5557	0–4	Mossel Bay	A crèche Sibling of 5488	
5578	0–4	Mossel Bay	Unknown	CVA9
4886	5–9	Mossel Bay	B crèche	CVA9
4876	5–9	Mossel Bay	Unknown	
4869	0–4	Mossel Bay	Unknown	CVA9
5488	5–9	Mossel Bay	A crèche Sibling of 5557	
1606	0–4	Mossel Bay	C crèche	
1607	5–9	Mossel Bay	B crèche	
1608	0–4	Mossel Bay	B crèche	
1609	0–4	Mossel Bay	B crèche	

*typing by 5’UTR sequencing and BLASTn analysis; E5- echovirus 5; E6- echovirus 6; E9- echovirus 9; E15- echovirus 15; E16- echovirus 16; E30-echovirus 30; CVA9- coxsackie virus A9; CVB3- coxsackie virus B3

**Fig. 1:**
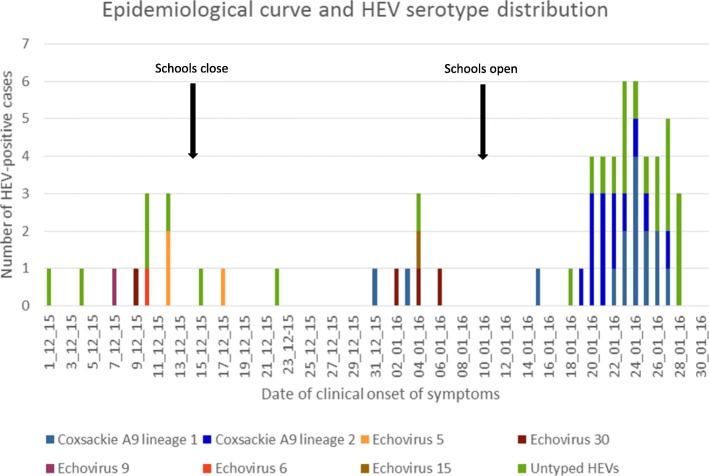
Epidemiological curve of HEV-positive cases in Mossel Bay by date of symptom onset and serotype distribution, 1st December 2015 to 30th January 2016 (*n* = 63)

Epidemiological investigations showed that 3 crèches (A, B and C) were linked to 26 outbreak cases: 6 cases from crèche A, 16 cases from crèche B and 4 cases from crèche C. Of the remaining 17 cases, two were family members of crèche-related cases, and no definitive link to a crèche could be established for 15 cases. The epidemiological curve illustrating the background sporadic cases and progression of the outbreak is shown in Fig. 1.

**Fig. 2:**
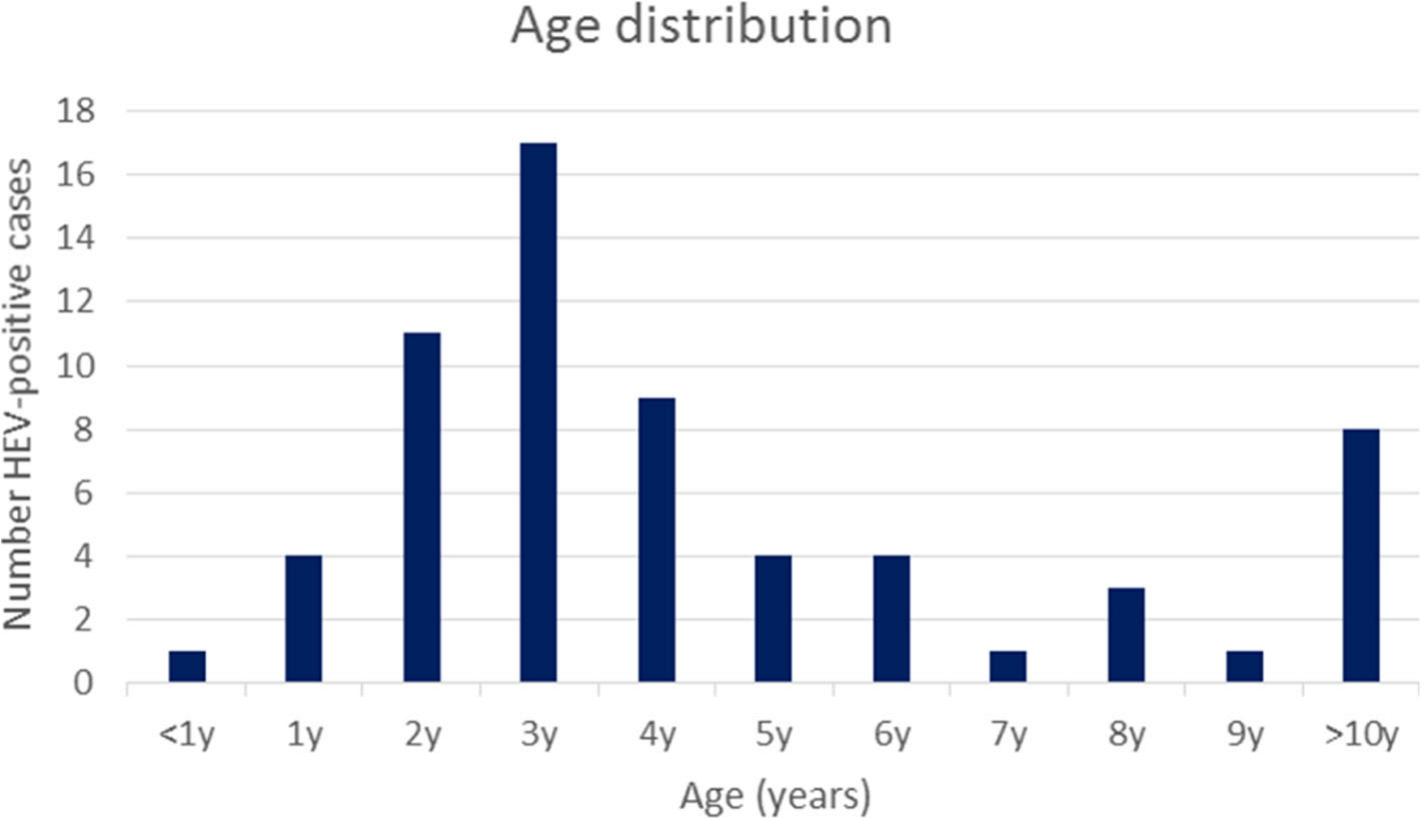
Age distribution of HEV- positive aseptic meningitis cases (n = 63)

The age of confirmed HEV-positive cases ranged from 10 days to 37 years with a median age of 3 years 9 months (Fig. 2). There was a predominance of males affected (66.7%; 42/63).

### HEV typing

#### VP1/VP2 typing

CSF was available for typing in 51/63 cases (81%), of which the VP1 or VP2 region could be successfully amplified in 29 samples (56.9%), with 24/29 (82.8%) identified as HEV species B. The PCR products of the remaining samples were too weak to sequence. No HEV species A and C were detected. The Enterovirus Typing Tool and BLASTn analysis identified the majority of samples to be CVA9 (*n* = 19; 79.2%) followed by echovirus 5 (n = 3; 12.5%) and one case each of echovirus 30 and echovirus 9 (4.2%).

#### 5’UTR typing

**Fig. 3:**
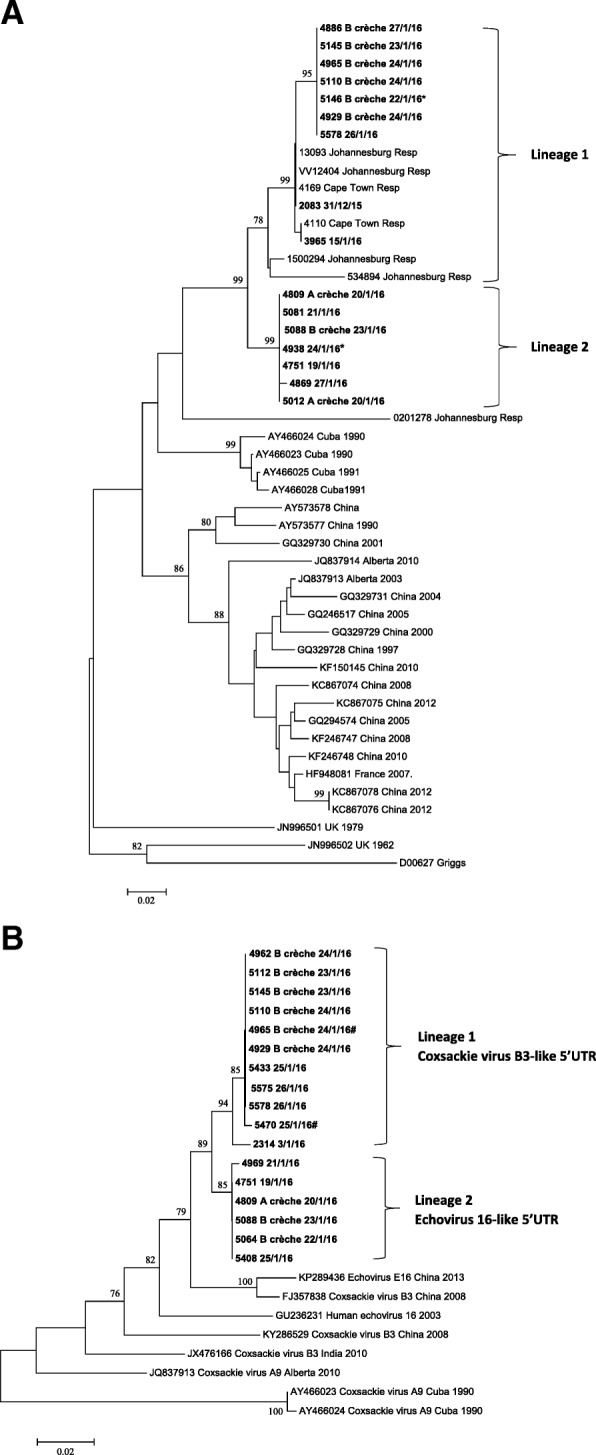
(**a**) VP1 and (**b**) 5’UTR phylogenetic trees of aseptic meningitis cases associated with CVA9

The 5’UTR primers were used to amplify samples negative for HEV species amplification (*n* = 22). Thirteen of the 22 (59.1%) cases were successfully amplified, sequenced, and BLASTn analysis identified 6 CVB3 cases, 3 echovirus 16 cases, 2 echovirus 30 cases and one each of echovirus 15 and echovirus 6 (Table 1 and Fig. 1). Interestingly, 8/9 CVB3 and echovirus 16 positives were detected during the outbreak period. Further amplification and sequencing of the 5’UTR region from known CVA9 samples (typing based on VP1/VP2 analysis) and associated with the outbreak, showed that these samples either had a CVB3-like or echovirus 16-like 5’UTR suggesting that these may be recombinant viruses. The assumption was therefore made that the samples identified as CVB3 and echovirus 16 by 5’UTR sequencing were in fact CVA9. This is supported by phylogenetic analysis of the VP1 and 5’UTR (Fig. 3A and B).

### Phylogenetic analysis

Phylogenetic analysis of the VP1 gene of CVA9 cases showed that the South African sequences clustered together and formed 2 lineages with a bootstrap value of 99%. Lineage 1 clustered with sequences from contemporaneous infections from Cape Town and Johannesburg. However, 7/9 Mossel Bay sequences formed a distinct cluster. These included 6 cases from the crèche B and 1 case with an unknown epidemiological link.

Lineage 2 contained only Mossel Bay sequences. They were from cases from crèches A and B, as well as patients with unknown epidemiological links (Fig. 3A). Both lineages co-circulated during the outbreak (Fig. 1 and Fig. 3A). Interestingly, 2 siblings, one who attended B crèche and the other a 10 day-old baby, had CVA9 that clustered with lineage 1 and lineage 2, respectively (Fig. 3A with asterisk).

The phylogenetic tree of the 5’UTR region (Fig. 3B) also showed 2 lineages, as found in the VP1 phylogenetic tree (Fig. 3A). Lineage 1 samples had a CVB3-like 5’UTR, while lineage 2 had an echovirus 16-like 5’UTR (as determined by Blastn analysis).

### Sequence analysis of Coxsackie virus A9 lineages

#### 5’ UTR and VP1 nucleotide sequences

**Fig. 4:**
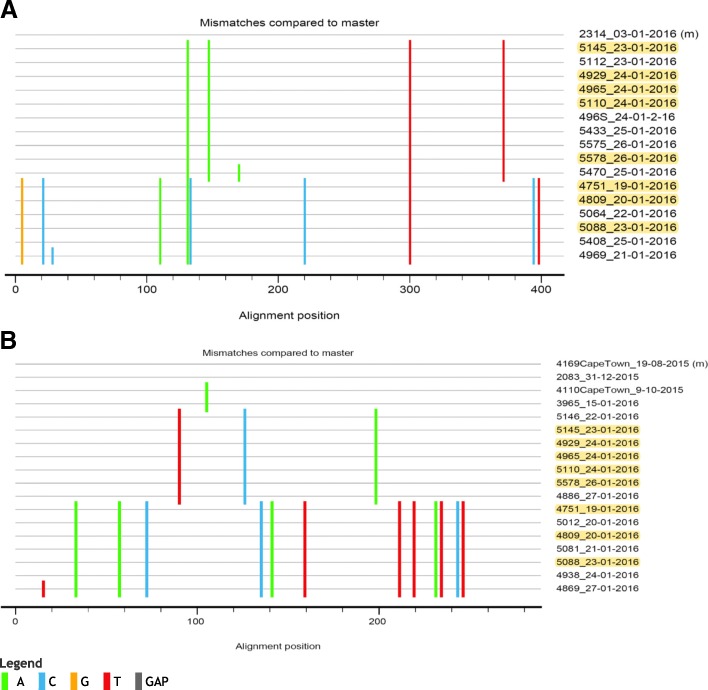
Highlighter plot of the 5’UTR (**a**) and VP1 (**b**) nucleotide sequences of CVA9-positive aseptic meningitis cases by similarity. Highlighted sequences are common to both data sets

The 5’UTR and VP1 nucleotide differences between the two lineages of CVA9 were compared using Highlighter plot. Lineage 2 had 8/417 (1.9%) and 13/289 (4.5%) nucleotide differences in the 5’UTR and VP1 region, respectively compared to lineage 1 (Fig. 4A and B). All nucleotide changes in the VP1 region were synonymous.

#### VP1 amino acid sequences

**Fig. 5:**
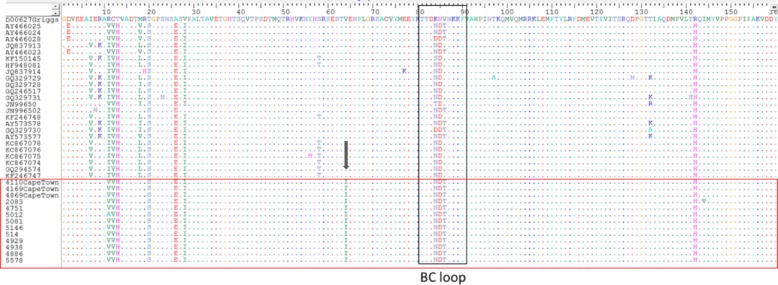
VP1 amino acid alignment of CVA9 –positive aseptic meningitis cases with other sequences from GenBank. Boxed sequences in black represent the BC loop and red box indicates South African sequences. Grey arrow shows unique amino acid change at position 64

The VP1 amino acid sequences were identical for both lineages 1 and 2 and were also identical to the isolates from Cape Town (Fig. 5). All Mossel Bay sequences had a unique isoleucine at position 64, while valine was present at this position in all other reference sequences, including the Griggs prototype (Fig. 5). A threonine replaced a valine within the BC loop (Fig. 5). The BC loop is one of them most exposed regions of the VP1 capsid protein.

## Discussion

In this study we describe the molecular epidemiology of HEV associated with a large outbreak of aseptic meningitis in a small coastal town in the Western Cape Province, South Africa which occurred in the summer of 2015/2016. During the 2-month period (December 2015 and January 2016) numerous cases presented to hospital with aseptic meningitis, of which 63 were confirmed by a private diagnostic laboratory to be caused by an HEV. Enterovirus typing of CSF samples collected over this period showed the presence of multiple circulating HEV species, including echovirus 5, 6, 9, 15 and 30 in the weeks preceding the outbreak. As 60% of the cases that occurred during the outbreak period were typed as CVA9, it was presumed that this was the causal agent of the outbreak. The first CVA9 case presented on 31st December 2015 and a further 2 cases with no apparent epidemiological links presented 3 and 15 days later. However, a dramatic increase in cases was noted within a week after crèches and schools reopened (13th January 2016). It can be postulated that the return of children to a crowded environment may have facilitated the spread of the virus.

Crèches and preschool facilities are vulnerable to rapid spread of viruses transmitted via the faecal-oral route, including HEVs [26–28]. The maintenance of good personal and communal hygiene practices with the prompt disposal of faeces and soiled clothing, disinfection of contaminated surfaces and the frequent washing of hands may be challenging in such an environment. In this outbreak three crèches with aftercare facilities were affected, recording numerous cases of aseptic meningitis. Although these facilities were not closed, in order to contain the outbreak, local health authorities initiated daily hand hygiene practise training for staff and parents, and children suspected of being ill were kept at home. While the primary route of transmission of HEV is via the faecal-oral route, other transmission routes including respiratory droplet spread and indirect spread through water- borne sources could also have played a role, although none of these were investigated in this study [29–31].

There are numerous reports of CVA9 as a significant causal agent of aseptic meningitis from around the world, including Japan (1961) [32], Northern Ireland (1970) [33], Poland (1974) [34, 35], Cuba (1990–1991) [36], Argentina (2007–2008) [37], Switzerland [38], Canada (2010) [39, 40], China (2006–2012) [41] as well South Africa [14]. Consistent with our study, the majority of cases occurred in children under 10 years of age.

Reasons for large outbreaks of specific HEV serotype-associated aseptic meningitis are not clear. Herd immunity plays an important role, and a decline in immunity to a particular serotype is either due to the emergence of a new serotype lineage or the re-emergence of a lineage from the past to which the population is susceptible. In addition, HEVs are subject to recombination and genetic divergence and these events may change the virus antigenicity, thus changing the immune response and also the HEV circulation patterns [42, 43]. Tracking of circulating HEV serotypes in South Africa has only recently recommenced with the growing interest in understanding serotype prevalence over time and clinical association. Hellferscee et al. [44] identified 33 different serotypes circulating over a 3-year period (2012–2014) in respiratory samples with echovirus 30, coxsackie virus B5 and enterovirus D68 most prevalent, and 3 cases of CVA9 detected. Unpublished data from Cape Town from 2014 to 2017 showed 38 different serotypes circulating in respiratory samples with different serotypes predominating in each year (Smuts, unpublished). Similarly, over the same period, more than 10 different serotypes were detected in the CSF of patients with central nervous system disease each year, with echovirus 9, echovirus 30 and CVA9 most commonly found (Smuts, unpublished). As historic sequence data of the VP1 region of CVA9 from South Africa is not available, it is not possible to determine if this outbreak was a result of an increase in the number of susceptible individuals in the population or the emergence of an immune escape mutant. However, it can be speculated that the homology of the outbreak virus with that found in Cape Town and Johannesburg early in 2015 would suggest that the virus had been in circulation for a while and that herd immunity may have dropped to below a critical threshold.

Nucleotide sequence and phylogenetic analysis of the 5’UTR and VP1 regions of CVA9 showed the co-circulation during this outbreak of 2 distinct lineages. Lineage 1 included the earliest aseptic meningitis cases detected on 31st December 2015, 3rd and 15th January 2016. This lineage also clustered with CVA9 from respiratory samples collected in Cape Town and Johannesburg earlier in 2015. The outbreak virus however, formed a distinct cluster within this lineage.

The VP1 amino acid sequence, on the other hand, was identical in both the outbreak viruses as well as viruses from Cape Town earlier in 2015. All had a unique isoleucine at position 64 which was not present in any of the reference sequences from GenBank, including the prototype Griggs strain. There was also an amino acid substitution in the hypervariable BC loop with a threonine replacing a valine. The VP1 capsid protein is 292 amino acids in length with many of the immunodominant serotype-specific epitopes located in the exposed BC loop (amino acid 82–93) [45, 46]. Although valine and threonine are structurally very similar, the substitution at this important neutralizing immunogenic site may have changed the conformation of the BC loop, thus altering the antigenicity. This may account for the increased susceptibility in this population. While the amino acid sequence of the VP1 gene was the same, the 2 lineages had distinct (synonymous) signature nucleotide substitutions in both 5’UTR and VP1 genes.

There are some limitations to this study. Firstly, the number of outbreak-related cases is likely under-reported. In the early stages of the outbreak all children with clinical manifestations suggestive of aseptic meningitis were routinely admitted to hospital where lumbar punctures were performed. However, as news of this outbreak spread it is possible that clinicians were familiar with the syndromic manifestations and opted for out-patient management of milder cases and not subject them to lumbar punctures. Anecdotally it was reported that many adults presented with a similar constellation of symptoms, but no CSF was collected and they were treated symptomatically. In this report CSF was available from only 1 adult, the father of a child attending one of the affected crèches. Attempts were made to obtain stool samples from adults presenting with compatible symptoms where the clinician had opted not to collect CSF, but no samples were received. Secondly, medical history and additional clinical data which would have provided additional information on the clinical course of aseptic meningitis was not available. A third limitation was the use of the 5’UTR for serotyping in some cases. As mentioned previously this is not the ideal target for serotyping, but where VP1 amplification failed, sequencing of this region did provide useful phylogenetic data. The 5’UTR sequence data showed that in the weeks preceding the outbreak echovirus 6, 15 and 30 were circulating in the Mossel Bay area. Further, correlation of the VP1 CVA9 sequence data with the 5’UTR results showed that there were 2 distinct lineages of this virus circulating during the outbreak. However, despite these shortcomings the study identified CVA9 as the causal agent in this large outbreak of aseptic meningitis.

## Conclusions

Our study identified CVA9 as the pathogen responsible for a large outbreak of aseptic meningitis, with the co-circulation of 2 distinct lineages.

## Additional files

Additional file 1: Fasta-formatted file of the 5’UTR sequences used in the identification of HEV serotypes and in the construction of the phylogenetic tree and analysis of the outbreak. (TXT 10 kb)

Additional file 2: Fasta-formatted file of the VP1 sequences used in the identification of HEV serotypes and in the construction of the phylogenetic tree and analysis of the outbreak. (TXT 11 kb)

## Abbreviations

5’UTR: 5′ untranslated region
CSF: Cerebrospinal fluid
CVA9: Coxsackie virus A9
CVB3: Coxsackie virus B3
E16: Echovirus 16
HEV: Human enterovirus
RT-PCR: Reverse transcription polymerase chain reaction
VP1: Viral capsid protein 1
VP2: Viral capsid protein 2
